# Therapeutic potential of mesenchymal stem cell‐derived extracellular vesicles: A focus on inflammatory bowel disease

**DOI:** 10.1002/ctm2.70075

**Published:** 2024-11-03

**Authors:** Laura Clua‐Ferré, Roger Suau, Irene Vañó‐Segarra, Iris Ginés, Carolina Serena, Josep Manyé

**Affiliations:** ^1^ Germans Trias i Pujol Research Institute IGTP Inflammatory Bowel Diseases Badalona Spain; ^2^ Hospital Universitari Joan XXIII Institut d'investigació sanitària Pere Virgili Tarragona Spain; ^3^ Centro de Investigación Biomédica en Red Madrid Spain

**Keywords:** Crohn's disease, exosomes, nanomedicine, ulcerative colitis

## Abstract

**Background:**

Mesenchymal stem cell‐derived extracellular vesicles (MSC‐EVs) have emerged as key regulators of intercellular communication, orchestrating essential biological processes by delivering bioactive cargoes to target cells. Available evidence suggests that MSC‐EVs can mimic the functions of their parental cells, exhibiting immunomodulatory, pro‐regenerative, anti‐apoptotic, and antifibrotic properties. Consequently, MSC‐EVs represent a cell‐free therapeutic option for patients with inflammatory bowel disease (IBD), overcoming the limitations associated with cell replacement therapy, including their non‐immunogenic nature, lower risk of tumourigenicity, cargo specificity and ease of manipulation and storage.

**Main Topics Covered:**

This review aims to provide a comprehensive examination of the therapeutic efficacy of MSC‐EVs in IBD, with a focus on their mechanisms of action and potential impact on treatment outcomes. We examine the advantages of MSC‐EVs over traditional therapies, discuss methods for their isolation and characterisation, and present mechanistic insights into their therapeutic effects through transcriptomic, proteomic and lipidomic analyses of MSC‐EV cargoes. We also discuss available preclinical studies demonstrating that MSC‐EVs reduce inflammation, promote tissue repair and restore intestinal homeostasis in IBD models, and compare these findings with those of clinical trials.

**Conclusions:**

Finally, we highlight the potential of MSC‐EVs as a novel therapy for IBD and identify challenges and opportunities associated with their translation into clinical practice.

**Highlights:**

- The source of mesenchymal stem cells (MSCs) strongly influences the composition and function of MSC‐derived extracellular vesicles (EVs), affecting their therapeutic potential. Adipose‐derived MSC‐EVs, known for their immunoregulatory properties and ease of isolation, show promise as a treatment for inflammatory bowel disease (IBD).
- MicroRNAs are consistently present in MSC‐EVs across cell types and are involved in pathways that are dysregulated in IBD, making them potential therapeutic agents. For example, miR‐let‐7a is associated with inhibition of apoptosis, miR‐100 supports cell survival, miR‐125b helps suppress pro‐inflammatory cytokines and miR‐20 promotes anti‐inflammatory M2 macrophage polarisation.
- Preclinical studies in IBD models have shown that MSC‐EVs reduce intestinal inflammation by suppressing pro‐inflammatory mediators (e.g., TNF‐α, IL‐1β, IL‐6) and increasing anti‐inflammatory factors (e.g., IL‐4, IL‐10). They also promote mucosal healing and strengthen the integrity of the gut barrier, suggesting their potential to address IBD pathology.

## Graphical abstract

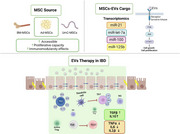

## 1 INTRODUCTION

Inflammatory bowel disease (IBD) is a heterogeneous disorder characterised by chronic inflammation of the gastrointestinal tract. Primary clinical phenotypes, including Crohn's disease (CD) and ulcerative colitis (UC), feature recurrent episodes of inflammation interspersed with periods of remission.^1^ CD can involve the entire gastrointestinal tract and cause transmural lesions, whereas UC causes more superficial lesions that are limited to the colon. Nevertheless, UC and CD share much of the same therapeutic armamentarium, and the same surveillance is recommended for both diseases to achieve therapeutic goals.^2^ As a chronic inflammatory disease, IBD poses significant challenges to healthcare systems worldwide, requiring a thorough understanding of its aetiology and the development of innovative therapeutic approaches.

The pathogenesis of IBD is multifactorial and involves a complex interplay between genetic predisposition, environmental factors and gut microbiota dysbiosis.^1^ This interplay leads to immune dysfunction and disruption of intestinal mucosal homeostasis, resulting in chronic inflammation and tissue damage. Key contributors to this homeostatic disturbance include compromised epithelial barrier function, altered innate immune cell activity and dysregulated lymphocyte responses within the intestinal lamina propria (the intraepithelial connective tissue between the epithelium and the muscularis mucosae) and mesenteric lymph nodes. The epithelial barrier serves as the first line of defense against luminal bacteria, and breaches can initiate a cascade of immune responses. Specifically, pathogenic bacteria or damage to the epithelial barrier activate dendritic cells (DCs), which migrate to the mesenteric lymph nodes and promote the differentiation of naïve T cells into either regulatory T cells (Tregs), which produce anti‐inflammatory cytokines, or various T helper (Th) cell subsets (such as Th1, Th2 and Th17), which secrete pro‐inflammatory cytokines.^3^

**FIGURE 1:**
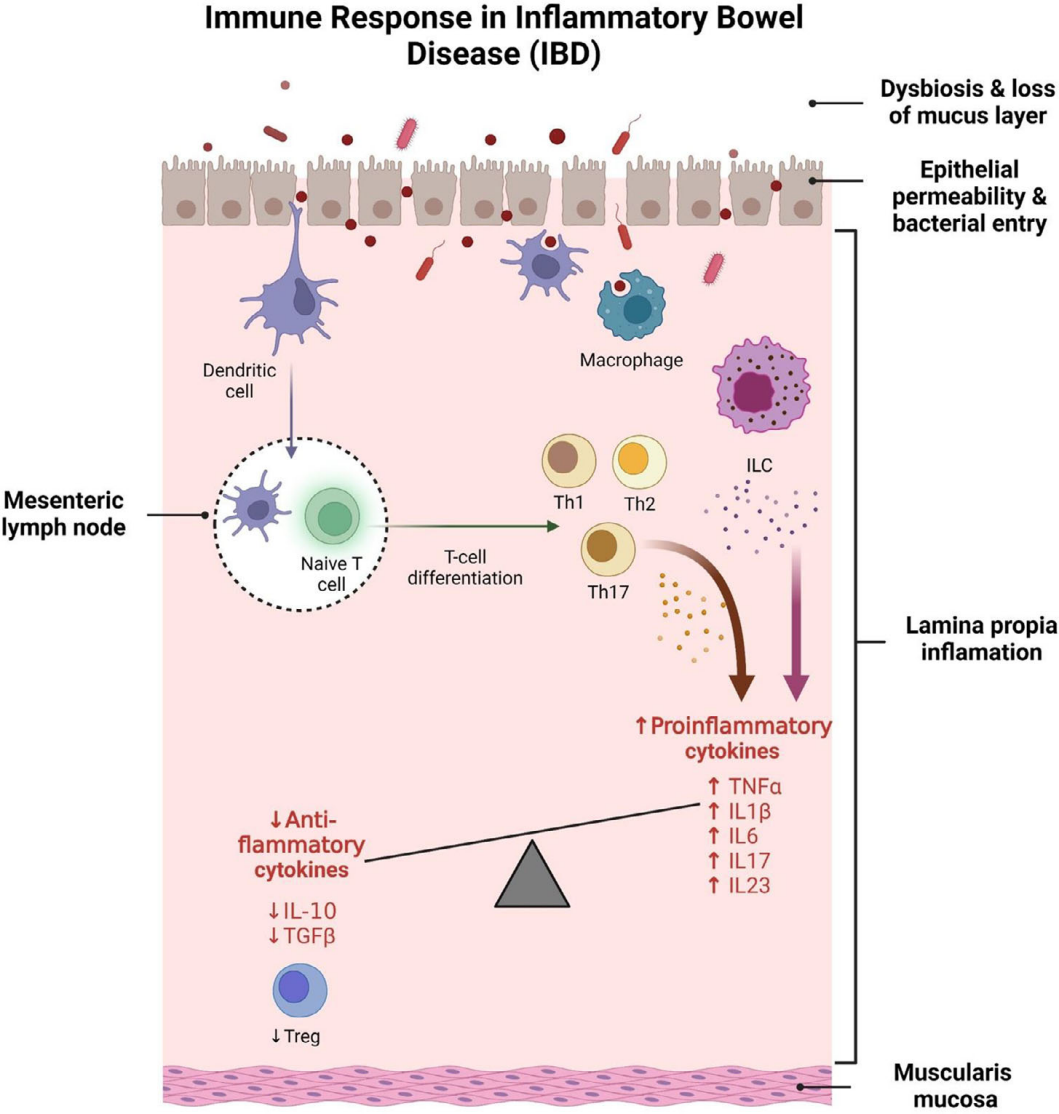
Immune dysregulation and barrier dysfunction in the pathogenesis of inflammatory bowel disease (IBD). The disruption of intestinal homeostasis in IBD is driven by dysbiosis in the gut lumen, loss of the mucus layer, and increased epithelial permeability, allowing bacterial translocation into the lamina propria. Breaches in the epithelial barrier activate dendritic cells, which migrate to mesenteric lymph nodes and promote the differentiation of naïve T cells into pro‐inflammatory T helper (Th) cell subsets. Under healthy conditions, regulatory T cells (Tregs) maintain immune homeostasis by secreting anti‐inflammatory cytokines such as IL‐10 and TGF‐β. In IBD, this balance shifts toward pro‐inflammatory Th cells, resulting in elevated levels of cytokines including TNF‐α, IL‐1β, IL‐6, IL‐17 and IL‐23. Additionally, dysregulated innate lymphoid cells (ILCs) exacerbate IBD by producing IL‐23, which stimulates IL‐17 secretion, further driving inflammation.

Under steady‐state conditions, Tregs are critical for suppressing excessive immune responses and maintaining tolerance to self‐antigens and commensal bacteria. They accomplish this by secreting anti‐inflammatory cytokines, such as IL‐10 and transforming growth factor beta (TGF‐β), which help dampen inflammation and promote tissue repair. A delicate balance between Tregs and Th17 cells is essential for effective immune defense without excessive inflammation.^4^ However, this balance is disrupted in IBD, favouring Th17 cells and leading to the overproduction of pro‐inflammatory cytokines such as IL‐17, IL‐22 and tumor necrosis factor alpha (TNF‐α), which perpetuate chronic inflammation. CD is associated with a skewed Th1/Th17 response, characterised by elevated levels of Th1‐related cytokines including IL‐12 and interferon gamma (IFN‐γ), whereas UC is predominantly associated with a Th2 response, characterised by increased expression of cytokines such as IL‐5, IL‐13 and IL‐4^5^ (Figure 1).

Recent studies have highlighted the role of innate lymphoid cells (ILCs) in maintaining intestinal health and their involvement in IBD pathogenesis.^6^, ^7^, ^8^ Similar to Th17 cells, certain ILCs produce IL‐23, which stimulates IL‐17 secretion and contributes to inflammation.^9^ Dysregulation of ILC activity has been linked to both the onset and the persistence of IBD, underscoring their role in early antimicrobial defense, tissue repair and regulation of the immune response.

Pro‐inflammatory cytokines such as IL‐1β, TNF‐α, IL‐6 and IL‐17 play a key role in the inflammatory response in IBD: IL‐1β contributes to immune cell recruitment, barrier disruption and Th17 differentiation^10^; TNF‐α, a critical mediator in IBD, also promotes mucosal barrier defects and perpetuates chronic inflammation^11^, ^12^, ^13^, ^14^; IL‐6 facilitates Th17 differentiation and supports Th1 persistence at sites of inflammation, exacerbating the chronic nature of the disease^15^, ^16^; and IL‐17, which is predominantly produced by Th17 cells, is central to the inflammatory cascade, and elevated levels are observed in the inflamed mucosa of patients with IBD.^17^, ^18^, ^19^ TGF‐β and IL‐10, while generally anti‐inflammatory, show dysregulated signalling in IBD, and fail to adequately counterbalance the pro‐inflammatory environment.^20^

Despite additions to the therapeutic armamentarium for IBD over the past decade, approximately half of patients will require at least one surgical intervention within 10 years of diagnosis.^21^ Thus, given the pronounced infiltration of innate and adaptive immune cells into the lamina propria of the intestinal mucosa during active IBD, there is a concerted effort among researchers and clinicians to develop treatments that efficiently modulate the immune response in IBD.^22^ Accordingly, the therapeutical goal is to promote immune regulation and facilitate tissue repair to reduce the severity of the disease, particularly its tendency to relapse.

Accumulating evidence highlights the ability of mesenchymal stem cells (MSCs) to secrete several bioactive mediators capable of modulating immune and inflammatory responses, and promoting intestinal tissue regeneration.^23^, ^24^ Consequently, therapeutic interventions based on MSC transplantation represent a promising strategy for diseases with an immunological basis including IBD. This is supported by the considerable therapeutic gains observed in numerous preclinical studies^25^ focused on IBD and in selected clinical trials^26^ involving patients with active CD. Unfortunately, despite promising results in clinical trials, MSC therapy remains predominantly in the experimental phase due to significant limitations, including safety concerns, inadequate cell survival, immune rejection and high cost. By contrast, MSC‐derived extracellular vesicles (EVs), which closely mimic the biophysical properties of MSCs, are considered to be potentially safer and more effective than MSCs themselves.^27^

The objective of the present review is to discuss the known therapeutic effects of MSC‐EVs that make them invaluable in the context of IBD. We provide a comprehensive exploration of the methods used to purify and characterise MSC‐EVs, highlighting their advantages and limitations. In addition, we examine the approaches employed to characterise the identity and purity of isolated EVs (Graphical Abstract). Recognising the clinical potential of MSC‐EVs will be crucial in advancing our understanding of basic cellular mechanisms and harnessing their therapeutic potential to treat IBD across various clinical scenarios.

## 2 FROM MSC THERAPY TO EXTRACELLULAR VESICLES IN IBD

### 2.1 State of the art of MSC therapy in IBD

MSCs are the most widely used cell type for cell transplantation‐based therapies because of their ability to differentiate into multiple lineages and the ease with which they can be isolated and expanded from different tissue sources.^28^, ^29^ In clinical trials, MSC‐based therapies have been validated in refractory perianal fistulas in patients with CD who are unresponsive to treatments including conventional drugs (antibiotics and immunomodulators), biologic therapy based on anti‐TNF antibodies (e.g., infliximab, adalimumab and certolizumab) and surgery. In 2018, Panés et al. completed a randomised, double‐blind, placebo‐controlled phase III trial using a single intrafistular injection of 120 × 10^6^ allogeneic MSCs, in which 56.3% of patients achieved remission that was sustained 52 weeks.^30^ A recent meta‐analysis on CD perianal fistulas reported that MSC therapy increased the healing rate compared with the control, achieving an overall healing rate of 64.1%.^31^ Furthermore MSCs have been administered intravenously or intraperitoneally to patients with refractory intraluminal inflammatory IBD, and have shown potential to alleviate clinical symptoms and improve mucosal lesions.^32^

However, it is important to note that the cure rates were not particularly high in the aforementioned clinical studies, and the long‐term efficacy of this therapy in maintaining remission remains unclear. As shown in a long‐term study of patients with perianal fistulas treated with MSCs, the results suggest that the cure rate at 2 years does not exceed 50%.^33^ Consequently, the clinical application of MSCs and their sustained therapeutic efficacy are limited by several constraints. Among these are obstacles pertaining to the viability of cells following transplantation, often accompanied by significant mortality due to immune rejection and suboptimal microenvironmental conditions including the absence of vascularisation and innervation.^34^ Additionally, determining the optimal cell dosage and frequency of administration represents a significant challenge. Moreover, the predominant route of administration, intravenous infusion, has been demonstrated to be an ineffective method due to the insufficient targeting of MSCs to the inflamed intestine. Indeed, a study in 2015 showed that less than 1% of MSCs injected intravenously successfully home to the damaged intestinal tissue.^35^ Consequently, although MSC therapy was initially believed to have potential in mitigating the effects of IBD, its efficacy may be constrained by the aforementioned limitations.

### 2.2 From cell therapy to vesicle therapy

Recent studies have shown that the immunoregulatory and regenerative properties of MSCs are primarily mediated through intercellular interactions facilitated by paracrine signalling. This process occurs via the MSC‐secretome—a collection of bioactive factors secreted by MSCs into their extracellular environment.^36^, ^37^ Due to the challenges related to the viability of MSC‐based therapies, there has been a shift in recent years toward using the MSC‐secretome or EVs therapies instead of direct MSC transplantation.

The MSC‐secretome consist of a variety of biocomponents that are secreted by a cell into its extracellular microenvironment. These components serve as effective mediators, either directly activating target cells or stimulating neighbouring cells to secrete active factors. In the context of IBD, it has been demonstrated that administration of the MSC‐secretome in experimental mouse colitis maintains the mechanical barrier, ameliorates clinical features and histological damage scores, and reduces inflammation.^38^, ^39^

**FIGURE 2:**
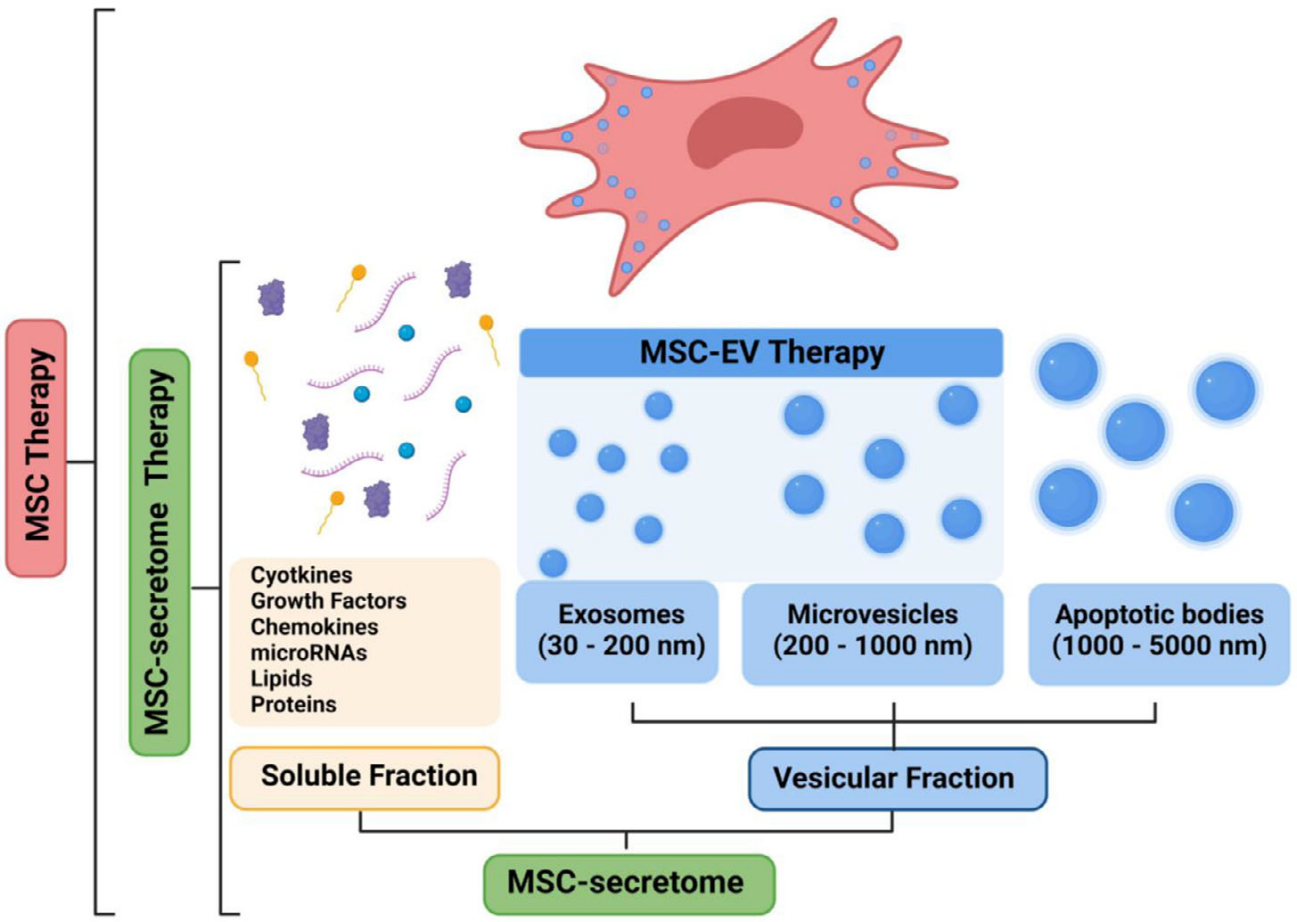
Mesenchymal stem cell (MSC) secretome. This figure depicts the diverse bioactive components secreted by MSCs, collectively referred to as the MSC‐secretome. The secretome is divided into two primary fractions: the soluble fraction, which includes proteins and soluble factors such as cytokines, growth factors and chemokines; and the vesicular fraction, which encompasses exosomes, microvesicles and apoptotic bodies. Exosomes (30‒200 nm) are generated through endocytosis, microvesicles (200‒1000 nm) are released directly from the plasma membrane via budding, and apoptotic bodies (1000‒5000 nm) are large vesicles formed during apoptosis.

The secretome can be divided into two primary fractions: the soluble fraction, which includes factors such as cytokines, growth factors, chemokines, microRNAs (miRNAs), lipids and proteins; and the vesicular fraction, which comprises exosomes, microvesicles and apoptotic bodies. Of these, only exosomes and microvesicles are used in MSC‐EV therapy (Figure 2).

The therapeutic potential of MSC‐based treatments raises the question of whether the effects are primarily driven by the MSC‐secretome as a whole or specifically by the MSC‐EV. Although both MSC‐EVs and the MSC‐secretome have demonstrated efficacy in experimental models of colitis, EVs offer several key advantages over the entire secretome. First, in contrast to the heterogeneous mixture of bioactive molecules present in the secretome, the MSC‐EV fraction offers greater specificity and control over its cargo.^40^ Second, the presence of non‐EV‐associated factors within the secretome may contaminate EVs preparations, whereas the homogeneity of MSC‐EVs minimises variability, ensuring more reproducible therapeutic outcomes and thereby enhancing both the efficacy and safety of EV‐based therapies. Third, a key advantage of EVs is their capacity to encapsulate therapeutic cargoes, facilitating the targeted transfer of such cargo to specific cells. This should result in enhanced therapeutic precision and a reduction in off‐target effects. Fourth, the modular nature of EVs permits surface membrane modifications that can be designed to improve targeting specificity and delivery efficiency. Indeed, in the future, identifying these specific therapeutic molecules will facilitate the engineering of EVs to precisely deliver targeted payloads, such as miRNAs or proteins, to specific cells. For these reasons, our review focuses on EV therapies instead of secretome‐based therapies.

Furthermore, in comparison to MSC therapy, which relies on live cells, MSC‐EV therapy offers a non‐cellular alternative that addresses several limitations of cell‐based treatments. Specifically, EV‐based therapy circumvents issues such as the need for vascularisation and the potential risk of tumorigenicity, thereby offering a safer and more streamlined approach.^41^ Sixth, the inherent immune compatibility of MSC‐EVs mitigates the risk of adverse immunogenic reactions, thereby increasing their applicability across diverse patient cohorts.^42^ Collectively, these attributes position MSC‐EVs as a promising avenue in regenerative medicine and drug delivery, offering an improved safety profile and broader patient accessibility compared with conventional cell‐based approaches. Table 1 highlights the key advantages of MSC‐EV therapy over MSC therapy.

**TABLE 1:** Comparison of the advantages of extracellular vesicles (EVs) therapy versus mesenchymal stem cell (MSC) therapy.

Aspect	MSC therapy	EV therapy
Safety concerns	Differentiation potential into various cell types.	Cell‐free compounds with no proliferative capacity.
Risk of immunogenicity	Possibility of immune response against transplanted cells, requiring immunosuppression.	Lower risk of immune rejection due to the lack of cell surface antigens.
Overcoming biological barriers	Limited ability to penetrate certain barriers.	Efficiently navigates through biological barriers.
Homing capacity	Limited, particularly when administered systemically.	High capacity for targeting specific tissues and organs, facilitated by surface molecules.
Viability	Need for vascularisation. Loss of viability.	Extended periods without loss of viability.
Therapeutic stability	Sensitive to changes in the local microenvironment.	Less susceptible to environmental changes.
Specificity	Secretes a nonspecific repertoire of molecules and biological factors (secretome) such as EVs, proteins, growth factors, angiogenic factors, hormones, cytokines, extracellular matrix proteins and proteases.	Exhibits specificity in cargo. The selective packaging ensures that EVs carry bioactive cargo tailored to their intended biological functions.
Storage and transport	Requires specialised storage conditions, making transportation more complex.	May be freeze dried for long‐term storage and easy transportation.

*Note*: EVs therapy demonstrates several advantages, including a lower risk of tumor formation, reduced immunogenicity, simpler administration and distribution, longer shelf‐life, stable phenotype, specific cargo delivery and easier management.

EVs have unique properties that allow them to efficiently reach target tissues. They are coated with specific biomolecules allowing their internalisation by target cells, and they possess inherent homing capabilities that allow them to target specific tissues and organs and shield them from degradation.^43^ Targeted delivery of EVs is facilitated by the interaction between surface molecules and receptors expressed on recipient cells at the target site. As a result, EVs can effectively cross biological barriers and reach the intended site, thereby maximising therapeutic efficacy while minimising off‐target effects.^44^ By contrast, MSCs have a lower homing capacity, especially when administered systemically, resulting in suboptimal tissue targeting.^45^ In addition, the cargo carried by EVs remains stable over time, ensuring more precise and controlled effects compared with MSC therapy, as well as higher reproducibility. Indeed, MSCs have been shown to have dual functionality, acting as either anti‐ or pro‐inflammatory agents depending on the host environment. By contrast, MSC‐EVs have shown consistent beneficial effects, indicating more predictable behaviour, a safer therapeutic profile and a higher therapeutic efficacy compared with their cells of origin.^46^ Finally, EVs can be freeze‐dried or lyophilised for long‐term storage, making them easier to transport and store compared with stem cells. This stability facilitates the development of “off‐the‐shelf” therapies that are readily available for clinical use. The convergence of versatility, safety, and precise efficacy underscores the potential of MSC‐EVs as a promising frontier in regenerative medicine, offering a new avenue for therapeutic innovation. Moreover, these attributes highlight MSC‐EVs as the key driver of the therapeutic effects of MSCs, offering a promising alternative to cell‐based therapies by harnessing the therapeutic potential of MSC‐derived factors without the risks associated with cell transplantation.

### 2.3 Classification and biogenesis of extracellular vesicles

EVs are bilayer vesicular nanoparticles found in the full spectrum of body fluids. They are classified into three main categories based on their size and biogenesis: exosomes, microvesicles and apoptotic bodies. Exosomes, with diameters ranging from 30 to 200 nm, result from the internalisation of extracellular components by the cell through endocytosis.^47^ This process involves wrapping of the cell membrane by invagination, resulting in the formation of endosomes. The endosomes then undergo maturation during which the membrane invaginates to produce intraluminal vesicles within the endosome. These intraluminal vesicles contain various internalised molecules such as antigens, nucleic acids, proteins and metabolites. A specialised endosome containing numerous intraluminal vesicles is called a multivesicular body (MVB). Ultimately, MVBs release their intraluminal vesicles into the extracellular environment.^48^ Microvesicles, also called ectosomes or microparticles, are EVs between 200 and 1000 nm in diameter that are released into the extracellular environment by direct budding or shedding from the plasma membrane. Finally, apoptotic bodies, which are larger than 1000–5000 nm in diameter, consist of relatively large fragments of cells containing organelles, histones, fragmented DNA and non‐coding RNAs from cells undergoing apoptosis. These vesicles are destined for clearance by phagocytosis.^49^ Given the diversity of EVs types and the ambiguity surrounding their biogenesis, the Minimal Information for Studies of Extracellular Vesicles has adopted a practical classification system. This system categorises EVs into ‘large EVs’ (>200 nm) and ‘small EVs’ (<200 nm) to facilitate standardisation and comparability in EVs research.^50^

While EVs differ in how they are formed, they have all been shown to contain a wide variety of bioactive cargoes, including proteins, RNA transcripts, miRNAs and even DNA, which can be transported to other cells to facilitate intercellular communication.^51^ EVs can fuse to the plasma membrane of target cells and release bioactive ‘cargoes’ that can activate and regulate specific genes. The composition of EVs can vary depending on the state of the cell of origin, conferring specific functions and targeting to EVs.^49^ Importantly, EVs released from MSCs have shown to share biological activity with their parental cells, representing a paradigm shift from traditional cell therapy to a versatile acellular approach.^52^

## 3 EVS ISOLATION AND CHARACTERIZATION

### 3.1 EVs isolation

Many techniques have been developed for the isolation of EVs and have been extensively reviewed elsewhere.^53^, ^54^, ^55^, ^56^ These techniques are mostly based on the biophysical and/or biochemical characteristics of EVs, such as size, density, shape or specific surface markers. Notably, each isolation method has the potential to affect the structural integrity and functional activity of EVs. Furthermore, there is increasing evidence that different classes of EVs may encapsulate different cargo molecules and have different functionalities depending on the class (exosomes, MVs and apoptotic bodies).^57^ Therefore, careful selection of the most appropriate isolation approach is critical to ensure high purity, yield and structural and functional integrity of the isolated EVs.

Several preparatory steps are usually performed prior to the isolation of MSC‐EVs. First, MSCs are cultivated in serum‐containing culture medium until they reach 70%–80% confluence. Then, to remove any residual serum proteins that may contaminate and interfere with EVs isolation, the cells are extensively washed and cultured in serum‐free medium for typically 12–48 h. During this step, the cells may also be exposed to chemical or physical stimuli to induce specific properties in the MSC‐EVs (see Section 7). The conditioned medium, which contains the soluble components of the MSC‐EVs, is then collected by centrifugation to remove any dead cells or cellular debris. This is then followed by the selection of an appropriate isolation,^58^ which is critical to ensure the purity and specificity of EVs, and minimises contamination from other cellular components. As the isolation method can influence both the yield and the biological activity of EVs, affecting the overall efficacy of the therapy, maintaining the functional integrity of EVs is essential to achieve reliable and reproducible therapeutic effects. Currently, there are no standardised methods for isolating pure EVs preparations, as most techniques are insufficient to ensure complete EVs purification free from non‐EV contaminants. Among the available methods, size‐exclusion chromatography (SEC) is one of the most promising for purifying EVs from biological fluids, due to its ability to reduce the co‐isolation of contaminants and its user‐friendly, scalable nature.^59^ An overview of EVs isolation techniques is provided in Table 2. Later, we only discuss the three methods that have been used in preclinical studies for IBD, but all techniques are included in Table 2 since they may be relevant for future applications.

**TABLE 2:** Summary of techniques for isolating extracellular vesicles (EVs).

Technique	Principle	Advantages	Disadvantages
Differential centrifugation and ultracentrifugation	Based on the density, size and shape of EVs, using different gradients and centrifugal speeds and forces.	Simple, requires minimal sample pretreatment, suitable for large‐scale production of clinical‐grade MSC‐EVs.	Does not allow separation of different types of EVs, requires a large sample size.
Size exclusion chromatography	Uses a chromatographic column with a porous stationary phase made of polymers, separating EVs based on their size, which determines the elution time from the column.	Ensures efficient EVs purification, prevents contamination from soluble proteins, GMP‐compliant, scalable.	Limited by column capacity, potential loss of smaller EVs.
Ultrafiltration	Utilises nanomembranes with molecular weight cut‐offs (10–100 kDa) to separate EVs by size.	Reduces isolation times and costs compared with other techniques.	Lower yields and purities due to vesicle–membrane interactions; potential for pore clogging and EVs damage due to shear stress.
Immunoaffinity capture	Based on surface protein markers of EVs membranes (e.g., CD9, CD63, CD81, CD82), with antibodies immobilised on a substrate that bind to EVs expressing specific antigens.	High specificity and purity for particular EVs subtypes.	Expensive, applicable only with small sample volumes.
Precipitation	Uses hydrophilic polymers such polyethylene glycol to reduce solubility and precipitate EVs by lowering hydration.	High yield, preserves EVs integrity, affordable, rapid extraction, suitable for large‐scale applications.	Lower purity, potential protein impurities due to precipitation reagents, may interfere with downstream processes.
EV isolation kits	Employ various techniques, including precipitation, immunoaffinity, chromatography, and centrifugation, based on established principles.	User‐friendly protocols, accessible to labs with limited resources, does not require specialised equipment.	Variable purity, yield, and size distribution; relatively high cost; less suitable for high‐throughput sample processing.

*Note*: Each method is described with regards to its principle, advantages and disadvantages. This comprehensive overview highlights the diverse approaches available for EVs isolation and their respective suitability for different applications.

Abbreviation: GMP, good manufacturing practice; MSC‐EV, mesenchymal stem cell‐derived extracellular vesicle.

### 3.2 EVs characterisation

Characterisation of EVs is essential to analyse sample purity and ensure reproducibility of experiments. Several methods have been developed to analyse EVs, each providing unique insights into their biophysical and biochemical properties. Nanoparticle Tracking Analysis (NTA) allows quantification (particles/mL) and diameter size distribution analysis of EVs in solution, providing valuable information on their size and concentration.^60^ In situations where an NTA instrument is not readily accessible, the microBCA assay shows minimal variability in the protein concentration values of EVs. Additionally, the microBCA assay shows the strongest correlation with NTA particle counts over a wide range of vesicle concentrations.^61^

Another typical characterisation method is Western blotting, which allows the detection of specific proteins within EVs samples, including membrane vesicle biogenesis proteins such as Alix and TSG101, and protein cargoes such as the heat shock proteins HSP60, 70 and 90. EVs membranes are also rich in lipid raft structures composed of cholesterol, spingomyosin and ceramide.^62^ Other methods include transmission electron microscopy, which provides high‐resolution imaging of EVs morphology allowing visualisation of their spherical shape and lipid bilayer membrane, and flow cytometry, which allows for the quantitative analysis of EVs based on their surface protein markers, facilitating the characterisation of EVs subpopulations. Notably, exosomes derived from MSCs express MSC surface markers (CD44, CD73 and CD90) and EVs markers such as CD9, CD63 and CD81.^63^ These characterisation methods can be used to gain a better understanding of EVs composition, size, morphology and surface markers, thereby advancing our knowledge of the biology of EVs and their potential clinical applications.

## 4 DYNAMIC ROLES AND MECHANISMS OF EXTRACELLULAR VESICLES

### 4.1 Potential differences between MSCs from various tissues and their impact on MSC‐EV dynamics

Despite functional similarities among MSC‐EVs, those derived from different tissues, such as bone marrow (BM‐MSCs), adipose tissue (AT‐MSCs) and umbilical cord (UmC‐MSCs), exhibit distinct characteristics and molecular compositions. At the cellular level, it is well documented that BM‐MSCs are traditionally considered the gold standard for MSC therapy. This is due to their extensive evidence of regenerative capabilities and immunomodulatory effects, despite the invasive nature of their isolation.^64^, ^65^ By contrast, AT‐MSCs are more readily accessible and have demonstrated higher proliferative capacity and enhanced secretion of anti‐inflammatory cytokines compared with BM‐MSCs.^66^, ^67^ Finally, UmC‐MSCs are noted for their unique immunoprivileged properties,^68^ which reduce the risk of rejection and enhance homing capabilities to inflamed tissues.^69^

These inherent differences between MSC sources can significantly influence the composition and functional properties of MSC‐EVs, including their cargoes, thereby affecting their therapeutic efficacy. However, comparative research in this area remains limited, and only one study to our knowledge has evaluated differences between EVs secreted from these three tissue sources.^70^ The study concluded that BM‐MSC‐EVs showed superior regenerative abilities, AT‐MSC‐EVs played an important role in immune regulation and UmC‐MSC‐EVs were more effective in tissue repair. In the treatment of IBD, which is primarily characterised by immune dysregulation, AT‐MSC‐EVs may be particularly beneficial. Also, they can be isolated from a variety of sites and are generally more accessible.^71^

Understanding these differences is essential for optimising MSC‐EV‐based therapies for IBD and tailoring treatments to exploit the unique advantages of MSCs from different tissue sources. In the next section, we discuss the cargo components from different origins of MSC‐EVs and examine the specific signalling pathways that are activated and may play a critical role in modulating and potentially reversing the pathophysiology of IBD. Table 3 summarises and classifies all preclinical studies on IBD using MSC‐EVs from different sources. Their therapeutic effects are discussed in Section 5.1.

**TABLE 3:** Mesenchymal stem cell‐derived extracellular vesicle (MSC‐EV) transplantation in preclinical murine models of inflammatory bowel disease (IBD).

MSC source	Isolation method	Dosage	Animal model	Days of treatment	Delivery route	Cargo	Target	Results	Refs
Murine BM	DC and UltraC	1 × 10^9^ EVs/each	DSS‐induced (C57BL/6) mice	7	3 × i.p.	N.D.	N.D.	Decreased intestinal fibrosis, angiogenesis and increased intestinal expression of Mucin 5ac, suggesting improved epithelial function.	^46^
Murine BM	DC and UltraC	100 µg	TNBS‐induced (SD) mice	10	i.v.	miR‐146a overexpression	TRAF6 and IRAK1	Decrease of DAI and histological colonic damage. Suppression of production TNF‐α, IL‐6 and IL‐1β.	^120^
Murine BM	DC and UltraC	10 µg/each	TNBS‐induced (SD) mice chronic	42	42 × i.v.	miR‐200b overexpression	ZEB1 and ZEB2	Inhibition of colonic fibrosis. The expression of E‐Cad was increased and the expressions of vimentin and α‐SMA was decreased.	^121^
Murine BM	DC and UltraC	50, 100 and 200 µg	TNBS‐induced (SD) mice	7	i.v.	N.D.	NF‐κB p65	Decrease of DAI and histological colonic damage. Downregulation of pro‐inflammatory cytokines levels, inhibition of NF‐κB p65 signal transduction pathways, modulation of anti‐oxidant/oxidant balance, and moderation of the occurrence of apoptosis.	^122^
Murine BM	DC	200 µg	TNBS‐induced (C57BL/6) mice	7	i.v.	miR‐378a overexpression	GATA2	Decrease of DAI and histological colonic damage. Reduction of GATA2 expression.	^123^
Murine BM	DC and UltraC	50 µg/each	DSS‐induced (BALB/C) mice	7	7 × i.p.	N.D.	JAK‐STAT	Decrease of DAI and histological colonic damage. Increase in IL‐10 and TGF‐β levels and the decline in VEGF‐A, IFN‐γ, IL‐12, TNF‐α, CCL‐24 and CCL‐17 levels. Promotion of M2 macrophage polarisation.	^124^
Murine BM	EVs isolation kit	50 000 µg/each	DSS‐induced (BALB/C) mice	8	8 × i.p.	N.D.	CCL1/CCR8 axis	Decrease of DAI and histological colonic damage. Increase of the number of Treg cells in the spleens and levels of IL‐4. Suppression of IL‐1β, IL‐6 and IL‐17A.	^125^
Murine BM	DC and UltraC	200 µg	DSS‐induced (C57BL/6) mice	10	i.v.	miR‐125 overexpression	Stat3 axis	Decrease of DAI and histological colonic damage. Decreased ratio of Th17 cells with elevated Treg cells ratio. Decreased expression of Stat3 and p‐Stat3 to inhibit Th17 cell differentiation.	^126^
Murine BM	DC and UltraC	100 µg/each	DSS‐induced (SD) rat	8	2 × i.v.	EphB2 overexpression	JAK‐STAT pathway	Attenuation of intestinal mucosa inflammation and restored intestinal muscosal barrier by the inhibition of pro‐inflammatory cytokines and upregulation of anti‐inflammatory mediators. EphB2‐EVs demonstrated a robust immunomodulatory effect to restore immune homeostasis by modulating Th17/Treg balance and restraining STAT3 activation.	^127^
Human BM	N.D.	32 mg/kg	DSS‐induced (C57BL/6) mice	7	4 × i.v.	miR‐181a overexpression	Claudin‐1 and ZO‐1	Alleviation of experimental colitis by enhancing intestinal barrier function through exosomal miR‐181a. Decreased expression of inflammatory cytokines in serum, including TNF‐α, IL‐6, IL‐1b, IL‐17 and IL‐18.	^128^
Human BM	DC and UltraC	200 µg/each	DSS‐induced (C57BL/6) mice	22	2 × i.v.	N.D.	Metallothionein‐2	Decrease of DAI and histological colonic damage. Downregulation of inflammatory responses by reduction of metallothionein‐2, maintained intestinal barrier integrity, and polarised M2b macrophages.	^129^
Murine AT	EVs isolation kit	100 µg/each	DSS‐induced (C57BL/6) mice	5	3 × i.p.	N.D.	N.D.	Decrease of DAI and histological colonic damage. Decreased levels of IFN‐γ, TNF‐α, IL‐12 and IL‐17 and increased levels of TGF‐β, IL‐4 and IL‐10.	^130^
Murine AT	Filtration and DC	1 × 10^5^ EVs/each	DSS‐induced (C57BL/6) mice	7	3 × i.p.	N.D.	N.D.	Attenuated disease severity. Suppression of IL‐6 expression in colon tissue and reduction of IL‐6 protein levels.	^131^
Diff. adipocytes	DC and UltraC	60 µg/each	DSS‐induced (C57BL/6) mice	7	2 × i.v.	N.D.	N.D.	Decrease of DAI and histological colonic damage. Inhibition of CD4+ T‐cell proliferation, decreased IFN‐γ, IL‐17, inhibited the differentiation of Th1/Th17 cells, promoted Treg induction and enhanced TGF‐β, IL‐10.	^132^
Human AT	DC and UltraC	EVs from 1 × 10^6^ cells	DSS‐induced (C57BL/6) mice	4	i.p.	N.D.	JAK‐STAT pathway	Attenuated disease severity. Decreased colonic lymphocyte infiltration. Reduction of IL‐6, TNF‐α, IFN‐γ, IL‐17 or IL‐12 and increase in IL‐10.	^133^
Human AT	DC and UltraC	300 µg/each	DSS‐induced (C57BL/6) mice	8	3 × i.v.	N.D.	N.D.	Decrease of DAI and histological colonic damage. Reduction of pro‐inflammatory factors (IL‐1β, IL‐6, IL‐12, TNF‐α), and increase of anti‐inflammatory mediators (IL‐13).	^134^
Human UmC	Ultrafiltration and EVs isolation kit	1000 µg/each	DSS‐induced (BALB/C) mice	9	3 × i.v.	miR‐378 overexpression	NLRP3	Regulation of macrophage pyroptosis. Decrease of DAI and histological colonic damage. Suppression of secretion of IL‐18, IL‐1β and caspase‐1 cleavage target by the decreased expression of NLRP3 inflammasomes.	^135^
Human UmC	EVs isolation kit	200 µg	DSS‐induced (C57BL/6) and TNBS‐induced (BALB/C) mice	5	i.p.	N.D.	TSG‐6	Decrease of DAI and histological colonic damage. Downregulation of pro‐inflammatory cytokines, upregulation of anti‐inflammatory cytokine, modulation of Th2 and Th17 cell response.	^136^
Human UmC	DC and UltraC	400 µg/each	DSS‐induced KM mice	7	3 × i.v.	N.D.	N.D.	Attenuated disease severity. Expression of IL‐10 gene was increased while that of TNF‐α, IL‐1, IL‐6, iNOS and IL‐7 genes was decreased. Lower infiltration of macrophages in colon tissue.	^137^
Human UmC	Ultrafiltration and EVs isolation kit	1000 µg/each	DSS‐induced (BALB/C) mice	7	3 × i.v.	miR‐326 overexpression	NEDD8, NF‐κB	Inhibition of neddylation. Decrease of DAI and histological colonic damage. reduced expression level of pro‐inflammatory factors such as IL‐1β and IL‐6.	^138^
Human UmC	DC and UltraC	200 µg	DSS‐induced (C57BL/6) mice	7	i.p.	N.D.	N.D.	Decrease of DAI and histological colonic damage. Protein levels of TNF‐α, IL‐6 and IL‐17 were decreased, while IL‐10 and TGF‐β1 were increased.	^139^

Abbreviations: α‐SMA, α‐Smooth Muscle Actin; AT, adipose tissue; BM, bone marrow; DAI, disease activity index; DC and UltraC, differential centrifugation and ultracentrifugation; DSS, dextran sulphate sodium; N.D., not determined; NF‐κB, nuclear factor kappa B; NLRP3, NLR family pyrin domain containing 3; i.p., intraperitoneal injection; i.v., intravenous injection; SD, Sprague‒Dawley; STAT3, signal transducer and activator of transcription 3; TNBS, 2,4,6‐trinitrobenzene sulphonic acid; TRAF6, tumour necrosis factor receptor‐associated factor 6; Tregs, regulatory T cells; TSG‐6, TNF‐stimulated gene 6; UmC, umbilical cord; VEGF, vascular endothelial growth factor.

Over the past decade, researchers have focused on how pathological states and lifestyles affect the therapeutic properties of adipose‐derived MSCs. For example, conditions such as obesity^72^ and ageing^73^ significantly impair the therapeutic efficacy of these cells. In addition, CD itself affects the therapeutic properties of adipose‐derived MSCs, even in patients in remission and those with adipose tissue distant from the affected bowel, such as the subcutaneous fat depot.^74^, ^75^, ^76^ Similarly, it was recently shown that MSCs from healthy donors with a history of smoking (including ex‐smokers) promote macrophage polarisation to an M1 pro‐inflammatory phenotype, fail to inhibit T‐ and B‐cell proliferation in vitro, and enhance the expression of inflammatory markers, including IL‐1β.^77^ This is likely due to a pro‐inflammatory epigenetic signature induced by cigarette smoking, which compromises the therapeutic potential of adipose‐derived MSCs. Given these findings, the selection of the best donors for MSC isolation is critical to obtain EVs with optimal therapeutic properties such as strong immunomodulatory and regenerative capabilities, thereby enhancing their overall therapeutic potential.

### 4.2 Cargo characterisation of MSC‐EVs and its potential therapeutic effect in IBD

As the exact mechanisms by which EVs exert their therapeutic effect in IBD are still unknown, characterisation studies of the different cargoes are very important to better understand their role in immunity, barrier function and inflammatory pacification, especially as the EVs cargo is highly dependent on the MSC origin. Many studies have been conducted to fully characterise the cargoes of EVs derived from MSCs using transcriptomic, proteomic and lipidomic analyses, followed by bioinformatic and/or in vitro approaches to better understand the pathways involved in their therapeutic effect.

#### 4.2.1 Transcriptomics

Specific mRNAs and, more importantly, miRNA components of the cargo, have been shown to be enriched in MSC‐EVs of diverse origins. Interestingly, however, the miRNA repertoire in EVs does not strictly reflect that of the cells of origin. Instead, EVs selectively incorporate a variety of RNA species.^78^ There are several examples of miRNAs that are present in EVs independent of the cell of origin. Notable among these is miR‐21, which is highly enriched in MSC‐EVs of bone marrow,^78^, ^79^, ^80^, ^81^, ^82^ umbilical cord^83^, ^84^ and embryonic origin.^85^ miR‐21 has been reported to be involved in the enhancement of anti‐inflammatory M2 macrophage polarisation by regulating the TGF‐β inhibitor PTEN^85^ (Figure 1). Targeting M2 polarisation in IBD is likely to be beneficial, as these diseases typically show an imbalance in the M1/M2 equilibrium and are characterised by pro‐inflammatory M1 macrophage profiles.^86^ Interestingly, the TGF‐β signalling pathway, which is involved in the pathophysiology of IBD in processes such as chronic inflammation, fibrosis and mesenteric fat hyperplasia,^78^ has been bioinformatically identified to be inhibited by miRNA/mRNA MSC‐EV cargoes using pathway enrichment analyses.^79^, ^87^, ^88^, ^89^, ^90^ Furthermore, treatment of activated macrophages with bone marrow‐derived exosomes, which naturally contain miR‐21 (among others), exerted an immunomodulatory action by reducing IL‐6 and IL‐1β expression.^79^ Supporting this, a recent study, showed that IL‐6 activity in macrophages in vitro was strongly reduced upon treatment with bone marrow MSC‐EVs.^75^ Finally, the suppression of miR‐21‐5p profoundly affected the immunosuppressive action of MSC‐EVs.^90^

**FIGURE 3:**
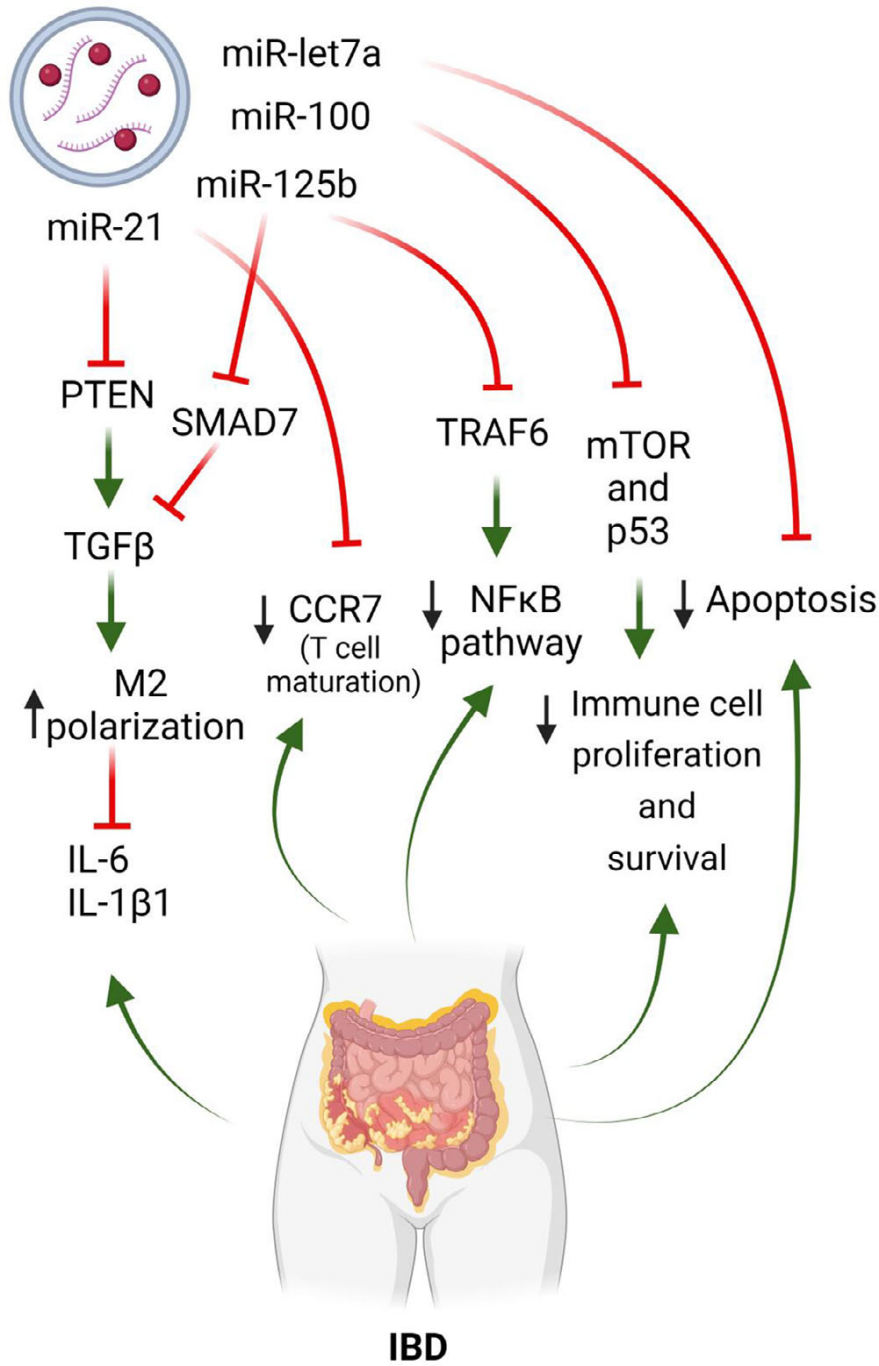
Mechanisms of mesenchymal stem cell‐derived extracellular vesicles and their therapeutic effects in inflammatory bowel disease. This figure illustrates the mechanisms through which mesenchymal stem cell‐derived extracellular vesicles exert therapeutic effects in inflammatory bowel disease. Key pathways include modulation of the immune response, reduction of inflammation, promotion of tissue repair and improvement of intestinal barrier function.

Several of the most important miRNAs identified in MSC‐EV cargoes are listed in Figure 3. Of these, miR‐21 has also been implicated in blocking CCR7,^91^ a chemokine key for gut homeostasis and bacterial tolerance, and for DC and T‐cell maturation and homing processes to lymph node and Peyer patches.^92^ Indeed, CCR7 has been observed to be upregulated in the lamina propria in CD^93^ and is also upregulated in Th1/Th17 cells in murine ileitis.^94^ Additionally, the mRNA of cytokines such as IL‐12A, IL‐17A and IL‐23A, which are involved in fate of CD4+ T cells to Th1 and Th17 populations, were found in EVs derived from perivascular^89^ and gingival.^88^ CD has been typically described as a Th1‐driven disease, with key involvement of Th17 cells,^95^ which has led to new therapies targeting the Th17/IL‐23 axis in IBD.^96^ Therefore, the presence of these mRNAs in EVs might not be beneficial for the treatment of IBD.

Among the many miRNAs identified in various EVs from different sources, other notable examples besides miR‐21 include miR‐125b,^79^, ^80^, ^81^, ^83^, ^84^, ^97^ miR‐100^79^, ^83^, ^84^, ^85^ and miR‐let‐7a.^84^, ^98^, ^99^ Although these miRNAs have not been directly implicated in IBD pathogenesis or recognised for their therapeutic or anti‐inflammatory effects in the disease, bioinformatic analyses have revealed their potential involvement in IBD‐related pathways. For example, miR‐125b‐5p targets TNF receptor‐associated factor 6 (TRAF6) and SMAD7, which are involved in the nuclear factor kappa B (NF‐κB) and TGF‐β signalling pathways, respectively. Importantly, the NF‐κB pathway was enriched or key in the miRNA cargoes of some EVs.^85^ One of the main orchestrators in IBD, TNF‐α, produced upon NF‐κB activation, has been extensively demonstrated in clinical settings to be important in IBD progression in many patients, although its inhibition is not curative.^100^, ^101^ In addition, the aforementioned cytokines, IL‐6 and IL‐1β, also depend on the activation of this pathway.

miR‐100‐5p targets genes such as mTOR and p53, which contribute to proliferation and cell survival, with the PI3K/Akt and MAPK pathways, by acting as an upstream regulator. Indeed, pathway enrichment analyses of the miRNA/mRNA cargo of EVs of bone marrow, adipose and endometrial origin, revealed the strong involvement of these vesicles in the PI3K/Akt pathway.^85^, ^87^, ^102^ More broadly, PI3K and MAPK pathways are involved in immune cell proliferation, but also in intestinal epithelial maintenance through processes such as autophagy, which is essential for the health of Paneth cells.^103^ Genetic variants of the autophagy gene ATG16L1 have also been implicated in the pathogenesis of CD and in intestinal antibacterial activity.^104^

Finally, miR‐let‐7a has been observed to target genes involved in apoptosis.^97^ An imbalance in intestinal epithelial cell death results in barrier permeability, leading to the chronic intestinal inflammation present in IBD. Excessive apoptosis later results in necroptotic cell death, which may also induce other lytic cell death mechanisms such as pyroptosis and ferroptosis, further increasing chronic inflammation.^105^

#### 4.2.2 Proteomics

The analysis of proteomic cargoes from MSC‐EVs in biological pathways is consistent with transcriptomic approaches. Results from several studies have revealed that protein cargoes are mainly enriched in immune system‐related processes, independent of cell origin, including pathways important for the therapeutic effectiveness of EVs such tissue reparation, angiogenesis, neurogenesis and wound healing.^105^, ^106^, ^107^, ^108^, ^109^, ^110^, ^111^

Proteomics analysis also revealed that EVs contain integrin‐ and cadherin‐related pathways involved in their extravasation to inflamed tissues,^106^, ^110^, ^112^ as well as chemokines implicated in cell migration.^109^, ^113^ In addition, and consistent with the transcriptomic cargo, the PI3K/Akt pathway was enriched in the protein cargoes, and many cytokines involved in cell survival, proliferation, Wnt pathways and immune cell differentiation (JAK/STAT) were also present.^106^, ^108^, ^110^, ^112^ Finally, the protein profiles from MSC‐EVs of different origin (induced pluripotent stem cells, gingival and bone marrow) were also enriched in cell adhesion and extracellular–matrix interactions such as focal adhesions.^109^, ^112^, ^113^, ^114^

#### 4.2.3 Lipidomics

Lipidomics has proven to be essential for characterising the cargo of EVs and predicting their therapeutic outcome. Two studies have performed lipidomic analyses, with both showing that MSC‐EVs are enriched in mainly saturated free fatty acids, cardiolipins, glycolipids^115^ and sphingomyelins.^82^ While cardiolipin has been described to be present only in the mitochondrial membrane, it might serve to stabilise the phospholipid membrane of EVs. Interestingly, anti‐cardiolipin antibodies have been identified as pro‐thrombotic elements in patients with IBD,^116^ and so the beneficial effect of this lipid in EVs is unclear. What is known is that prior supplementation of beneficial lipids in the in vitro culture of EVs completely changes their lipid profile. Indeed, unsaturated fatty acid supplementation has been shown to have pro‐revolving effects on inflammation in IBD.^117^ Sphingomyelin is a member of the sphingolipid family of lipids, which are essential for the intestinal cell membrane homeostasis, and sphingolipid metabolism has been shown to be dysregulated in IBD.^118^ In fact, sphingolipid metabolism is unbalanced towards the rapid production of inflammatory members of this family, with a loss of species such as ceramides or sphingomyelin.^119^ Thus, the incorporation of sphingomyelin from MSC‐EVs may have a beneficial effect in the affected intestine in IBD.

In conclusion, the proteomic, transcriptomic and lipidomic analysis of MSC‐EVs cargoes of different cell origins have revealed the presence of anti‐inflammatory, reparative and homeostatic components. These findings suggest that MSC‐EVs may provide therapeutic benefits to the inflamed intestine in diseases such as CD and UC.

## 5 THERAPEUTIC EFFECTS OF MSC‐EVS IN PRECLINICAL STUDIES OF IBD

In the preclinical studies outlined in Table 3, MSC‐EVs have demonstrated the ability to improve weight loss and colon shortening by attenuating intestinal mucosal inflammation and restoring the intestinal mucosal barrier. This effect is achieved by suppressing pro‐inflammatory cytokines, increasing the levels of anti‐inflammatory mediators, and strengthening the intestinal barrier. Table 3 is organised according to the type of cell source from which the EVs were derived, including bone marrow, adipose tissue and umbilical cord. Notably, no discernible differences associated with the different cell sources were observed in terms of reduction of histologic colonic damage and disease activity index (DAI) score, which is composed of change in body weight, diarrhoea, haematochezia and attenuated neutrophil infiltration. Nevertheless, as stated previously, MSC‐EVs derived from bone marrow are the most commonly used tissue source^46^, ^120^, ^121^, ^122^, ^123^, ^124^, ^125^, ^126^, ^127^, ^128^, ^129^ compared with adipose tissue^130^, ^131^, ^132^, ^133^, ^134^ and umbilical cord.^135^, ^136^, ^137^, ^138^, ^139^

### 5.1 Cell sources of MSC‐EVs in preclinical studies of IBD

#### 5.1.1 Bone marrow‐derived EVs

MSC‐EVs derived from bone marrow (BM‐MSC‐EVs) have demonstrated the ability to reduce the levels of pro‐inflammatory mediators including TNF‐α, IL‐6, IL‐17, IL‐1β and IFN‐γ while upregulating the expression of anti‐inflammatory factors and increasing the levels of proteins such as IL‐4, IL‐10 and TGF‐β.^46^, ^120^, ^121^, ^122^, ^123^, ^124^, ^125^, ^126^, ^127^, ^128^, ^129^ IL‐10 acts as a key regulator of the immune system by limiting excessive inflammatory responses that can lead to tissue damage. Moreover, IL‐10 plays a critical role in maintaining immune homeostasis, particularly within the gastrointestinal tract.^140^ Similarly, TGF‐β serves as a potent suppressor of inflammatory responses induced by luminal bacterial antigens, thereby contributing to the induction of immune tolerance.^141^, ^142^ Accordingly, MSC‐EV therapy may modulate the levels of these important factors and maintain mucosal homeostasis, thereby improving IBD symptoms.

In addition, recent reports note that miR‐125a and miR‐125b, which are enriched in BM‐MSC‐EVs, downregulate Th17 cell differentiation by inhibiting STAT3 expression, which is required for early Th17 cell development.^126^ Moreover, miR‐146 is a well‐known anti‐inflammatory miRNA, and EVs obtained from BM‐MSCs overexpressing this miRNA can regulate NF‐κB p65 phosphorylation and inhibit the expression of TRAF6 and IL‐1 receptor‐associated kinase 1 (IRAK1), thereby inhibiting the release of inflammatory factors in macrophages, and reducing colonic inflammation.^120^ A recent study has reported that BM‐MSC‐EVs containing miR‐181 promote the expression of claudin‐1 and ZO‐1, thereby strengthening intestinal barrier function.^128^

#### 5.1.2 Adipose tissue‐derived EVs

MSC‐EVs derived from adipose tissue (AT‐MSC‐EVs) share similar anti‐inflammatory properties with BM‐MSC‐EVs, including the attenuation of the same pro‐inflammatory cytokines, and additionally reduce the expression of IL‐12. Of note, IL‐12 plays a critical role in the promotion and maintenance of intestinal inflammation associated with IBD, mainly by promoting the differentiation of naïve T cells into Th1 effector cells.^130^, ^131^, ^132^, ^133^, ^134^

#### 5.1.3 Umbilical cord‐derived EVs

**FIGURE 4:**
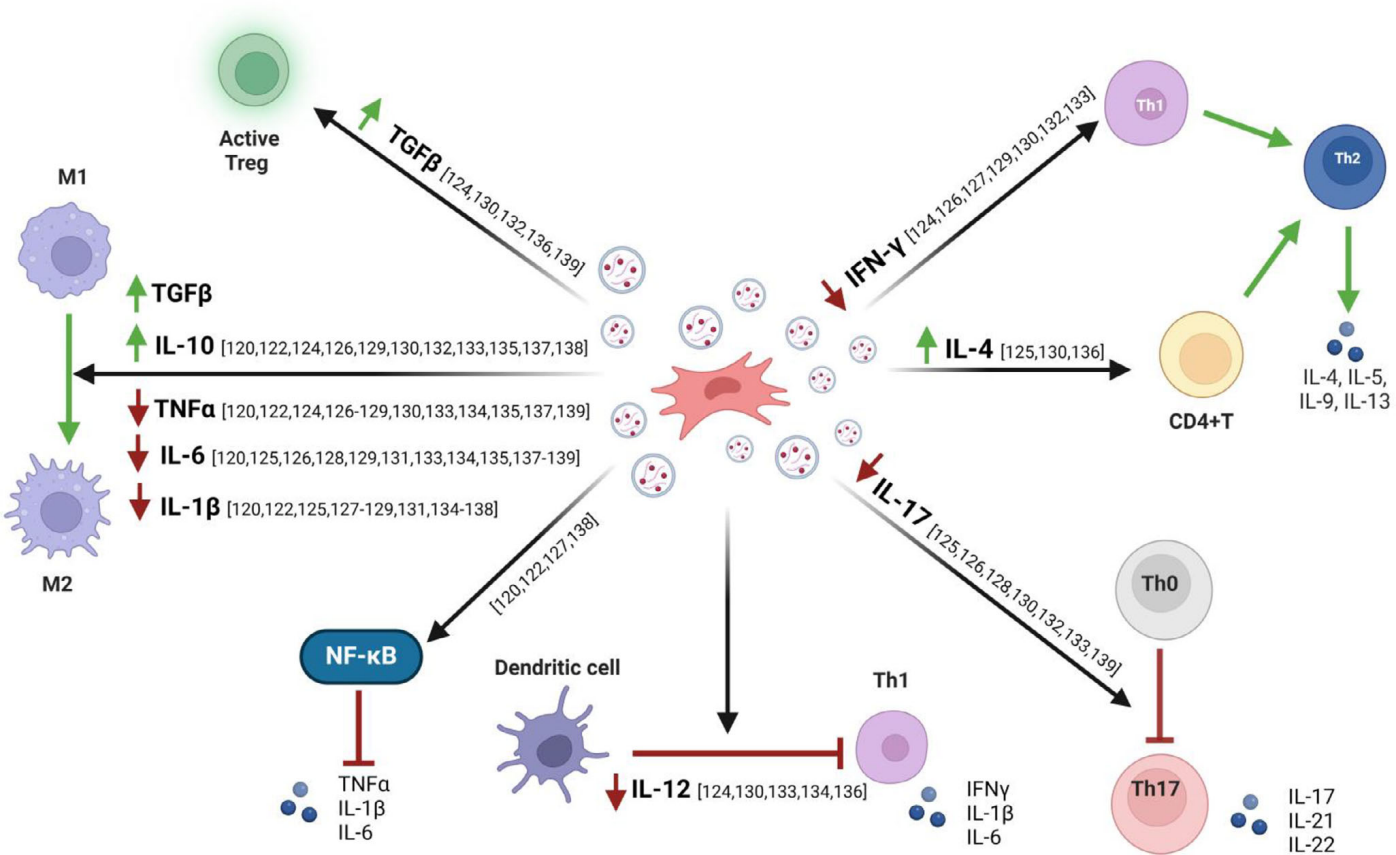
Therapeutic effects of mesenchymal stem cell‐derived extracellular vesicles in preclinical studies of inflammatory bowel disease. Illustration of the mechanisms of mesenchymal stem cell‐derived extracellular vesicles and their therapeutic effects on inflammatory bowel disease in preclinical studies, achieved by suppressing pro‐inflammatory cytokines and increasing the levels of anti‐inflammatory mediators.

Finally, MSC‐EVs derived from UmC (UmC‐MSC‐EVs), similar to BM‐ and AT‐MSC‐EVs, inhibit the levels of key pro‐inflammatory cytokines including TNF‐α, IL‐1β and IL‐6, but also, IL‐7.^135^, ^136^, ^137^, ^138^, ^139^ IL‐7 acts as a master regulator of T‐cell differentiation and stimulates the expression of cell adhesion molecules and monocyte chemoattractant protein (MCP)‐1, leading to increased immune cell infiltration into colonic tissue.^143^ As a result, targeting IL‐7 expression may inhibit immune cell migration and infiltration into colonic tissue, resulting in anti‐inflammatory effects. In addition, overexpression of miR‐378a in UmC‐MSC‐EVs suppresses the secretion of IL‐18, IL‐1β and caspase‐1 cleavage target by reducing NLRP3 inflammasome activity, preventing pyroptosis, and thus increasing cell survival in dextran sulphate sodium (DSS)‐induced colitis mice.^135^ A summary of the mechanisms of MSC‐EVs and their therapeutic effects on IBD are shown in Figure 4.

### 5.2 EVs isolation methodologies

Of the six EVs isolation techniques previously discussed, only three have been used in preclinical studies on IBD: ultracentrifugation, EVs isolation kits and filtration. The most commonly used technique to isolate MSC‐EVs is ultracentrifugation (61.9%), which involves a combination of different centrifugation and ultracentrifugation steps. While this method represents the gold standard among the EVs isolation methods, a major drawback of this technique is the inability to obtain a pure EVs sample, resulting in a highly heterogeneous sample that may yield less reproducible results. The second method reported in the use of EVs isolation kits (23.8%), which can provide satisfactory results in terms of sample purity. However, the main limitation of these kits is their high cost, which makes them unsuitable for processing large sample volumes.

### 5.3 EVs dosage

Preclinical studies in IBD have investigated a wide range of doses of MSC‐EVs, but there is currently no consensus on the ideal dosage. In addition, two distinct methods for quantifying the EVs administered and routinely used. The first method measures the amount of EVs in terms of the number of particles per millilitre using NTA analysis. With this method, the dose can range from 1 × 10^5^ to 1 × 10^9^ EVs, and can be administered as a single or multiple infusion (up to three). The second method is based on the protein quantification of the sample, with doses ranging from 10 to 50 000 µg per animal in single or multiple infusions (up to 42). This variability in measurement techniques makes it difficult to compare results across different studies, which are summarised in Table 3. Nevertheless, the most commonly used dose of MSCs is in the range of 10‒1000 µg per mouse, administrated in three different doses (Table 3). In line with FDA guidelines,^144^ the conversion from mouse to human doses is performed using a human equivalent dose equation, which incorporates dose adjustments based on both body weight and surface area. This approach estimates a human dosage range of 25 500‒255 000 µg/kg.

### 5.4 Chemical‐induced experimental colitis models

Over the last three decades, researchers have used various animal models to study intestinal inflammation and gain insight into the pathogenesis of IBD. However, it is important to recognise that no single animal model fully recapitulates the complex nature of human IBD. Preclinical studies investigating MSC‐EVs therapies for IBD have predominantly used chemically induced colitis models, such as those induced by DSS and 2,4,6‐trinitrobenzenesulphonic acid (TNBS), using mice and rats. These models have been shown to be effective in inducing intestinal inflammation, which typically manifests shortly after induction.^145^ Importantly, these models are technically feasible and have facilitated the study of genetic and immunologic mechanisms underlying IBD susceptibility during various phases of colitis development, including acute, recovery and chronic phases. Administration of DSS in drinking water at concentrations of 3%‒5% for 4–10 days results in acute injury characterised by partial crypt depletion and mucosal erosive lesions and accompanied by infiltration of mononuclear and polymorphonuclear cells. Macrophages and CD4+ T cells play a key role in wound healing within the lamina propria during the late recovery phase. Furthermore, repeated cycles of DSS administration can simulate the chronicity observed in human IBD. Similarly, rectal administration of TNBS combined with ethanol induces severe transmural colitis mediated by Th17 and Th1 immune responses.^146^

### 5.5 Administration routes of EVs

Intravenous and intraperitoneal administration are the most common routes of administering MSC‐EVs in preclinical studies. Although the former is the preferred route (59%). For intraluminal lesions, a local administration through mucosa may provide a more concentrated delivery to the inflamed tissue, but the retention time is short, resulting in less therapeutic benefit. Accordingly, encapsulation of MSC‐EVs with hydrogels may help to achieve spatio‐temporal control in vivo, thereby increasing the therapeutic efficacy of EVs. In this line, a recent study evaluated the effect of rectal administration of a mucoadhesive, and thermosensitive hydrogel loaded with the lyophilised secretome of MSCs in a DSS‐induced colitis mouse model. The Pluronic‐based hydrogel reduced body weight loss, gene expression of pro‐inflammatory factors and colonic tissue damage.^147^

Encapsulation of EVs in hydrogel biomaterials can increase their retention time at the target site and promote their continuous release, prolonging their therapeutic effect. The benefits of hydrogels has been recently studied for several different diseases, including type 1 diabetes,^148^ osteoarthritis,^149^ renal ischaemia,^150^ myocardial infarction,^151^ spinal cord injury^152^ and hindlimb ischaemia.^153^

## 6 MSC‐EVS THERAPY IN CLINICAL STUDIES

**FIGURE 5:**
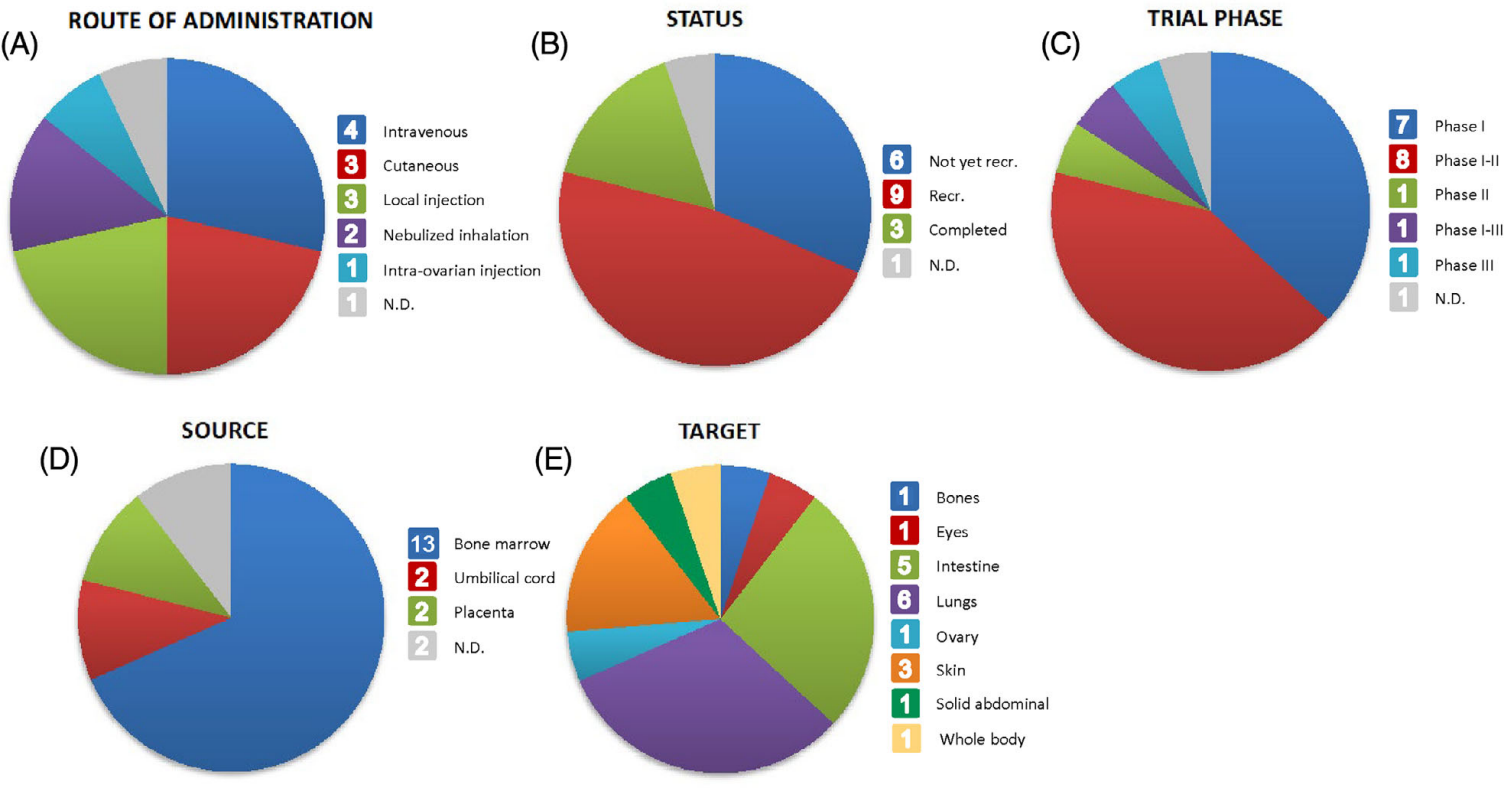
Representative results of the analysis of Table 3, summarising a total of 19 ongoing and completed clinical trials using mesenchymal stem cell‐derived extracellular vesicles. The number of trials corresponding to each section is displayed inside the boxes. (A) Route of administration of mesenchymal stem cell‐derived extracellular vesicles (MSC‐EVs). (B) Trial status provided by clinicaltrials.gov. (C) Trial phase provided by clinicaltrials.gov. (D) Source of MSC‐EVs used in each trial. (E) Organs targeted for administration of MSC‐EVs. N.D., not determined; Recr., recruiting.

Among the many applications of MSC‐EVs in clinical trials, we have focused on their role in immune regulation and tissue regeneration. By accessing the website www.clinicaltrials.gov and using the keywords ‘extracellular vesicles’ and ‘mesenchymal stem cells’, we obtained a set of studies that were reviewed and filtered according to their status and the information they provided. Clinical studies with incomplete information, in vitro or ex vivo studies, and withdrawn projects were removed, leaving a total of 19 research papers for comparison. As can be seen in Table 4 and Figure 5, most of these studies were conducted in adult humans of both sexes. Regarding the source of EVs, 68% of the trials use bone marrow, the preferred option, for harvesting, followed by 11% of the studies using placenta and 11% using umbilical cord. Regarding the phase and status of the different trials, most of them are in phase I or phase I–II (37% and 42%, respectively), 47% of them are still recruiting or have not yet started recruitment (32%), and only a few of them are completed. This shows that although the studies are promising and many of them have recruited a considerable number of participants, the use of MSC‐EVs in humans is still at an early stage of research and needs further investigation. All of these studies present a wider variety of targets depending on the disease they want to treat. Nevertheless, as shown in Figure 5, the top targeted organs are the lungs (32% of the studies), followed by the intestine and the skin (26% and 16%, respectively).

**TABLE 4:** Clinical trials utilising extracellular vesicles (EVs) derived from mesenchymal stem cells (MSCs) for the treatment of inflammatory bowel disease (IBD) and other inflammatory conditions.

MSC source	Age	Enr.	Dosage	Condition	Days treatment	Delivery route	Targ.	Ph.	Status	Brief description	Trial number
N.D.	18‒50	20	N.D.	Segmental bone tissue defects	N.D.	N.D.	Bones	1‒2	Not yet Recr.	Treatment of patients with segmental bone tissue defects using mesenchymal stem cells enriched by EVs.	NCT05520125
BM	>18	15	50 µg of EVs for single eye	Retinitis pigmentosa	1 dose	Intravitreal injection	Eyes	1‒2	Not yet Recr.	Safety and efficacy of intravitreal injection of GMP‐compliant BM‐MSC‐EVs in patients with retinitis pigmentosa.	NCT06242379
Placenta	18‒70	80	N.D.	Perianal fistula in patients with CD	1 dose	Local injection	Intestines	1‒2	N.D.	Safety of human placenta MSC‐EVs for treatment of perianal fistula in patients with CD.	NCT05499156
BM	18‒75	36	1 dose of 15 or 30 mL or 2 doses of 30 mL of ExoFlo (10^9^ EVs/mL	Perianal fistula, CD	1 or 2 doses (day 0 and 3 months)	Local injection	Intestines	1‒2	Recr.	Safety and feasibility of ExoFlo as a treatment for perianal fistulising CD.	NCT05836883
Placenta	18‒70	80	N.D.	Fistula perianal	1 dose	Local injection	Intestines	1‒3	Recr.	Safety of human placenta MSC‐EVs for treatment of complex anal fistula.	NCT05402748
BM	18‒75	10	15 mL of ExoFlo (10^9^ EVs/mL)	UC, IBD	15 doses (15 mL days 0, 2, 4; 30 mL from week 2 to 46)	i.v.	Intestines	1	Recr.	Adult allogeneic hBM MSC‐EVs isolate product, for the treatment of medically refractory UC.	NCT05176366
BM	18‒75	10	15/30 mL of ExoFlo (10^9^ EVs/mL)	CD, IBD	15 doses (15 mL days 0, 2, 4; 30 mL from week 2 to 46)	i.v.	Intestines	1	Recr.	Adult allogeneic hBM MSC‐EVs isolate product, for the treatment of medically refractory CD.	NCT05130983
UmC	18‒75	240	10^9^ particles/ml in 5 mL	COVID‐19‐induced lung injuries	5 days (2 doses per day)	Nebulised inhalation	Lungs	1	Recr.	Safety and efficacy of nebulised inhalation of MSC‐EVs combined with standard therapy for COVID‐19‐infected individuals.	NCT05787288
UmC	18‒80	80	5 mL of 10^9^ particles/mL	Long COVID‐19 syndrome	10 doses (2 per day)	Nebulised inhalation	Lungs	1	Recr.	Safety and effectiveness of MSC‐EVs nebulisation inhalation therapy for the treatment of chronic cough after COVID‐19 infection.	NCT05808400
BM	18‒85	81	10/15 mL ExoFlo (10^9^ EVs/mL)	Acute respiratory distress syndrome	1 dose	i.v.	Lungs	1‒2	Not yet Recr.	Safety and efficacy of i.v.‐administration of human MSC‐EVs derived from BM, ExoFlo, as treatment for acute respiratory distress syndrome.	NCT05127122
BM	18‒75	970	ExoFlo 15 mL of ExoFlo (10^9^ EVs per mL)	ARDS	1 dose	i.v.	Lungs	3	Recr.	Safety and efficacy of i.v.‐administration of hBM MSC‐EVs, ExoFlo, for the treatment of hospitalised patients with ARDS.	NCT05354141
BM	18‒85	102	10/15 mL ExoFlo (10^9^ EVs/mL)	COVID‐19, ARDS	1 dose	i.v.	Lungs	2	Comp.	Safety and efficacy of i.v.‐administration of hBM MSC‐EVs, ExoFlo, as treatment for moderate‐to‐severe acute respiratory distress syndrome.	NCT04493242
BM	3‒14 days (children)	3	20, 60 and 200 pmol phospholipid/kg body weight of UNEX‐42	High risk for bronchopulmonary dysplasia	1 dose	i.v.	Lungs	1	Not yet Recr.	A safety study of i.v. stem cell‐derived EVs (UNEX‐42) in preterm neonates at high risk for bronchopulmonary dysplasia.	NCT03857841
BM	20‒38	10	2 mL of EVs from MSCs (30 million cells)	Premature ovarian failure	1 dose	Intraovarian injection	Ovary	1‒2	Recr.	Safety and effectiveness of intraovarian injection of MSC‐EVs in patients with Premature Ovarian Failure diagnosis.	NCT06202547
BM	>18	10	10^7^ EVs particles for each cm^2^ treated area	Burns	3 weeks (1 dose/week)	Cutaneous	Skin	1	Recr.	Treatment of patients with deep second degree burns of the skin with hBM‐derived MSC‐EVs.	NCT05078385
BM	>6	10	Ascending dose of AGLE‐102 (MSC‐EVs)	Dystrophic Epidermolysis Bullosa	6 doses every 14 days	Cutaneous	Skin	1‒2	Not yet Recr.	Effectiveness and safety of AGLE‐102 on lesions in subjects with Epidermolysis Bullosa.	NCT04173650
N.D.	>21	10	100 µg MSC exosomes/g ointment	Psoriasis	20 days (3 doses per day)	Cutaneous	Skin	1	Comp.	Safety and tolerability of the application of the MSC‐EVs ointment with repeated topical application on adult healthy subjects.	NCT05523011
BM	18‒75	20	1 dose of ExoFlo (10^9^ EVs/mL)	Solid organ transplant rejection	1 dose	i.v.	Abdominal organ	N.D.	Avail.	ExoFlo for patients who have or might have severe or life‐threatening abdominal solid organ transplant rejection.	NCT05215288
BM	18‒85	60	ExoFlo 15 mL (10^9^ EVs/mL)	COVID‐19, postviral syndrome, dyspnoea	1 dose	i.v.	Whole body	1‒2	Not yet Recr.	Safety and efficacy of i.v. administration of human hBM MSC‐EVs, ExoFlo, as treatment for post‐acute COVID‐19 and chronic post‐COVID‐19 syndrome.	NCT05116761

Abbreviations: ARSD, acute respiratory distress syndrome; BM, bone marrow; CD, Crohn's disease; Enr, enriched; GMP, good manufacturing practice; hBM, human bone marrow; i.v., intravenous; N.D., not determined; Ph, phase; Recr., recruiting; Targ., target; UC, ulcerative colitis; UmC, umbilical cord.

Regarding the route of administration, intravenous administration is used in 21% of the trials, indicating that it is the preferred option, followed by local injection and cutaneous administration, both used in 16% of the studies. When the mechanism of EVs delivery to the target is unknown or when the target organs are inaccessible, intravenous administration is a wise option. The dose of administration varies depending on the disease. In most studies, EVs are given in a single dose of 5–30 mL (10^7^–10^9^ EVs/mL), which is similar to the preclinical studies shown in Table 3, where concentrations of EVs are in the same order of magnitude (but the number of administrations is higher). There are two exceptions (trials NCT05176366 and NCT05130983) where the studies are focused on treating conditions related to IBD, and the number of administrations increased to 15 across 46 weeks. In this case, instead of acute treatment, the investigators aim to study the effect of long‐term treatment with EVs in medically refractory UC and CD.

In contrast to intravenous administration, a local injection ensures that the entire dose of EVs reaches the intended target while avoiding contact with other organs or tissues and possible side effects. Some tissues that might benefit from this route of administration include the eyes, skin, ovaries and colon. However, studies should be conducted to determine how direct injection affects the patient's health. While most local injections are given as a single dose, a few studies aimed at treating skin conditions have planned to apply many doses of EVs across a range of times including daily, weekly or every other week. This is of great interest in the context of lung‐related diseases such as COVID‐19 and the use of nebulised inhalators, as EVs can be integrated into this platform and reach the desired target via inhalation, thereby facilitating rapid and precise delivery. The clinical trials we found using these inhalators (NCT05787288 and NCT05808400) were related to COVID‐19 issues. In both cases, the researchers chose to administer a few of doses per day for 5 and 10 days, respectively, using a concentration of 5 mL of EVs (10^9 ^EVs/mL).

In general, while preclinical studies have shown that intravenous administration is the most common option for EVs delivery, clinical trials present a wider range of options that help to improve the delivery and specificity of therapeutic EVs.

## 7 MODULATION OF MSC‐EVS CARGOES AND PRODUCTION

### 7.1 Biochemical stimuli

Various techniques permit the modification of MSCs to either overexpress or underexpress certain factors, allowing MSC‐EVs to be tailored in their therapeutic potential. A common strategy in the engineering of MSC‐EVs is to expose the cells to biochemical stimuli during the preconditioning phase. Extensive research on the biochemical stimulation of MSCs has focused on exposing cells to a variety of inflammatory mediators, including TNF‐α, IFN‐γ, TGF‐β, IL‐1 family and lipopolysaccharide.^154^ This stimulation induces MSCs to produce a number of responsive biomolecules with significant functional utility, including various cytokines and chemokines (such as IL‐1β, IL‐6 and IL‐8), proteases and gelatinases (particularly metalloproteases), protease inhibitors and factors that promote angiogenesis and cell survival including vascular endothelial growth factor (VEGF), fibroblast growth factor (FGF)‐2, hepatocyte growth factor (HGF), insulin‐like growth factor 1 (IGF‐1), angiopoietin and MCP‐1.^155^ These molecules collectively contribute to immunomodulation, angiogenesis, tissue regeneration, and exhibit anti‐inflammatory, antifibrotic and neuroprotective effects.

### 7.2 Physical stimuli

Physical stimuli can also be utilised during the preconditioning phase to alter the composition of MSC‐EVs. Among these, hypoxic culture (O_2_ < 5%) is a common method aimed at enhancing the therapeutic potential of MSC‐EVs for tissue and organ regeneration. A hypoxic environment inhibits apoptosis in MSC‐EVs and increases the expression of angiogenic factors such as VEGF and basic FGF.^156^, ^157^, ^158^ Selecting the appropriate conditions for hypoxic exposure during preconditioning is essential, as different oxygen levels ranging from .1% to 5% O_2_ can variably influence the characteristics and functional abilities of MSC‐EVs.^158^

### 7.3 Spheroid 3D cultures

Scale‐up production of EVs is a major challenge for translational applications. Recent findings show that culturing MSCs as aggregates (spheroids) in 3D culture wave motion promotes EVs secretion and can enhance their therapeutic potential in different disease models.^159^ Cells in the body are constantly exposed to 3D mechanical forces. These forces are generally translated into biochemical signals through mechanotransduction, which can stimulate different signalling pathways and influence cell behaviour. While the majority of studies using mechanical forces have been to regulate stem cell differentiation, it is apparent that mechanical stimulation can also be used as a means to modulate the secretome.^154^ MSC spheroid culture has been shown to dramatically enhance the secretion of EVs about 6.7‐fold more than cells under two‐dimensional culture, with enhanced immunomodulatory potential.^160^ Furthermore, MSC spheroid culture can elevate the secretion of proangiogenic factors (VEGF, bFGF, HGF, angiogenin, IL‐11), anti‐inflammatory markers (IL‐1ra, granulocyte‐colony stimulating factor, prostaglandin E2) and antifibrotic molecules.^161^, ^162^, ^163^, ^164^ In summary, these studies provide a promising strategy for scalable production of high‐quality EVs from MSCs with enhanced therapeutic potential.

## 8 FUTURE CHALLENGES

Despite the rapid progress in EVs research, several challenges must be overcome before EV‐based therapies can be translated into clinical practice. The isolation, purification, storage and accurate characterisation of EVs remain complex processes requiring substantial resources, skilled personnel and standardised methodologies. Currently, there are diverse isolation techniques with no universally accepted protocols, which affects data interpretation and comparability. The International Society for Extracellular Vesicles attempted to address these issues through a guideline in 2018, providing specific technical guidance on suggested protocols and steps to follow to document specific functional activities.^165^ Another limitation to be overcome in the near future is the detailed profiling of EVs components to elucidate the mechanisms underlying EV‐based therapy. Understanding the diverse molecular cargoes within EVs, including proteins, lipids, RNAs and other bioactive molecules, is critical to uncovering how these vesicles mediate regenerative, immunomodulatory and anti‐inflammatory effects. Therefore, a thorough characterisation of EVs composition not only advances our fundamental knowledge of EVs biology but also promotes the development of more effective and targeted EV‐based therapies.

Finally, the use of EVs in clinical settings, despite the availability of sufficient potent EVs, has been hampered by the low rate of EVs uptake by recipient cells and rapid clearance from the circulation, which could critically affect the therapeutic properties.^166^ To overcome this limitation, biomaterials such as hydrogels have recently been used to deliver high doses of EVs to the target tissue.^147^, ^162^, ^167^, ^168^, ^169^, ^170^, ^171^, ^172^ Due to the long healing time of regenerating tissues, it is crucial to develop novel biocompatible scaffolds as carriers for the sustained release of EVs and maintenance of their bioactivity at the target site.^173^ In addition, hydrogels can be tailored to achieve stimuli‐responsive gelation, thereby facilitating their delivery and enabling void filling.^174^ However, further investigation into the properties of hydrogels, such as stiffness, absorptive capacity and biodegradability, are warranted to elucidate how these characteristics influence the behaviour and efficacy of EVs.

## 9 CONCLUSIONS

With their unique phospholipid bilayer, EVs can carry bioactive molecules to modulate the behaviour of recipient cells, including proteins, lipids and nucleic acids that play critical roles in intercellular communication and regulation of various cellular functions. In particular, MSC‐EVs have therapeutic potential by modulating cells to enhance tissue repair, suppress pathological inflammation and improve immune function, highlighting their therapeutic promise in a wide range of diseases. Given their anti‐inflammatory effects and their role in promoting angiogenesis to the regenerate intestinal barrier, EVs may be particularly useful in the treatment of chronic inflammation associated with IBD,

EVs can be isolated in a variety of ways, with SEC emerging as the most appropriate technique due to its ability to preserve EVs integrity. EVs can be characterised using standardised techniques, such as NTA, microBCA, flow cytometry, Western blotting and transmission electron microscopy. Regarding the cellular source for obtaining therapeutic EVs, BM‐MSC‐EVs are commonly used, and AT‐MSC‐EVs have shown a significant role in immune regulation. Furthermore, AT‐MSCs offer easier accessibility and have demonstrated a higher proliferative capacity, making them a promising alternative.

Preclinical studies in IBD models have shown that MSC‐EVs can reduce intestinal inflammation, promote mucosal healing, and strengthen gut barrier integrity, suggesting their potential to restore disease pathology and promote healing. Regarding the dose of EVs, the order of magnitude is similar in murine preclinical studies and in clinical trials. However, multiple doses are administered in preclinical models rather than a single dose in humans. To translate EVs therapy from preclinical models to humans, it will be clearly important to consider the differences in weight between rodents and humans, and increasing the concentration of EVs and the number of administrations could enhance therapeutic benefits. Effective EVs therapy in vivo is highly dependent on the route of administration. Local injection may enhance therapeutic benefit by overcoming the challenges of rapid EVs clearance and low accumulation in target tissues often observed with intravenous administration. In addition, encapsulation of EVs in biomaterials can reduce their rapid clearance by the immune system and improve their bioavailability. Encapsulating EVs in hydrogel allows for targeted delivery to specific tissues, improves precision in reaching desired sites, and allows for controlled release over time, ensuring a sustained therapeutic effect.

While the preclinical evidence is compelling, clinical research is still in its early stages. The promising preclinical data underscore the potential of EV‐based therapies to revolutionise IBD treatment. However, there are still challenges that need to be overcome, such as the strategy for scalable production of high‐quality EVs. Stimulating MSC cultures with inflammatory cytokines and culturing in 3D aggregates under hypoxic significantly enhances EVs secretion and confers improved immunomodulatory potential. Moving forward, it is essential to transition from preclinical to clinical trials and develop standardised protocols for EVs production and quality control to ensure consistency and safety. Overall, the promising preclinical data underscore the potential of EV‐based therapies to revolutionise the treatment of immunomodulatory diseases such as IBD, ushering in a new era of targeted and effective medical interventions.

## AUTHOR CONTRIBUTIONS

Laura Clua‐Ferré wrote the first draft and contributed to the writing of the manuscript. Roger Suau, Irene Vañó‐Segarra and Iris Ginés contributed to different parts of the writing and reviewed the manuscript. Carolina Serena and Josep Manyé contributed to the writing and critically reviewed the manuscript.

## CONFLICT OF INTEREST STATEMENT

The authors declare they have no conflicts of interest.

## Data Availability

All data relevant to this study are included in the article. Additional data are available on request from the corresponding author.
